# A randomized controlled trial comparing different sites of high-velocity low amplitude thrust on sensorimotor integration parameters

**DOI:** 10.1038/s41598-024-51201-9

**Published:** 2024-01-12

**Authors:** Imran Khan Niazi, Muhammad Samran Navid, Christopher Merkle, Imran Amjad, Nitika Kumari, Robert J. Trager, Kelly Holt, Heidi Haavik

**Affiliations:** 1 Centre for Chiropractic Research, New Zealand College of Chiropractic, Auckland, New Zealand; 2 Faculty of Health & Environmental Sciences, Health & Rehabilitation Research Institute, AUT University, Auckland, New Zealand; 3 Department of Health Science and Technology, Aalborg University, Aalborg, Denmark; 4 Donders Institute for Brain, Cognition and Behaviour, Radboud University Medical Center, Nijmegen, The Netherlands; 5 Hamburg University of Applied Sciences, Hamburg, Germany; 6 Riphah International University, Islamabad, Pakistan; 7 Connor Whole Health, University Hospitals Cleveland Medical Center, Cleveland, USA; 8 College of Chiropractic, Logan University, Chesterfield, USA

**Keywords:** Neuroscience, Sensorimotor processing, Health care, Musculoskeletal system, Neurophysiology

## Abstract

Increasing evidence suggests that a high-velocity, low-amplitude (HVLA) thrust directed at a dysfunctional vertebral segment in people with subclinical spinal pain alters various neurophysiological measures, including somatosensory evoked potentials (SEPs). We hypothesized that an HVLA thrust applied to a clinician chosen vertebral segment based on clinical indicators of vertebral dysfunction, in short, segment considered as “relevant” would significantly reduce the N30 amplitude compared to an HVLA thrust applied to a predetermined vertebral segment not based on clinical indicators of vertebral dysfunction or segment considered as “non-relevant”. In this double-blinded, active-controlled, parallel-design study, 96 adults with recurrent mild neck pain, ache, or stiffness were randomly allocated to receiving a single thrust directed at either a segment considered as “relevant” or a segment considered as “non-relevant" in their upper cervical spine. SEPs of median nerve stimulation were recorded before and immediately after a single HVLA application delivered using an adjusting instrument (Activator). A linear mixed model was used to assess changes in the N30 amplitude. A significant interaction between the site of thrust delivery and session was found (F_1,840_ = 9.89, *p* < 0.002). Pairwise comparisons showed a significant immediate decrease in the N30 complex amplitude after the application of HVLA thrust to a segment considered “relevant” (− 16.76 ± 28.32%, *p* = 0.005). In contrast, no significant change was observed in the group that received HVLA thrust over a segment considered “non-relevant” (*p* = 0.757). Cervical HVLA thrust applied to the segment considered as “relevant” altered sensorimotor parameters, while cervical HVLA thrust over the segment considered as “non-relevant” did not. This finding supports the hypothesis that spinal site targeting of HVLA interventions is important when measuring neurophysiological responses. Further studies are needed to explore the potential clinical relevance of these findings.

## Introduction

A recent narrative review explored the effects of high-velocity, low-amplitude (HVLA) thrusts delivered to vertebral segments^1^. The article differentiated between HVLA thrusts applied to clinician chosen vertebral segment based on clinical indicators of vertebral dysfunction, in short segments considered as “relevant” vs HVLA thrusts delivered to predetermined vertebral segments not based on clinical indicators of vertebral dysfunction or segments considered as “non-relevant”^1^. Four of the eight included studies (i.e., 50%), which examined the effects of HVLA thrusts on segments considered as “non-relevant”, found a positive change in neuromuscular control measures^1^. In contrast, 14 of the 18 (i.e., 78%) of the studies which examined HVLA thrust to segment considered as “relevant” reported improvements in measures of neuromuscular control^1^. This raises the interesting question of whether the site of a HVLA thrust matters, and if so, how it might matter.

The term joint dysfunction is an umbrella term that can encompass anything from a infected joint to an arthritic join. However, for the purposes of this study the type of vertebral dysfunction we are talking about are the type of biomechanical lesions of the vertebral column that chiropractors and other manual therapists might apply their HVLA thrusts. Chiropractors do not usually randomly apply HVLA thrust at a segment of the spine. Usually, they will assess the spine for areas of the spine characterized by tight vertebral muscles, reduced intervertebral movement and tenderness to touch^2^. This type of joint dysfunction is often referred to as a vertebral subluxation in chiropractic profession^3–6^. Vertebral subluxation is a term recognised as biomechanical lesions of the vertebral column by the World Health Organization (ICD-10-CM code M99.1)^7^. However, other professions have used many other names, thus for the purposes of this study we will simply refer to it as joint dysfunction.

Neurophysiological measures are a novel and attractive means of examining the importance of applying HVLA vertebral thrusts to a segment considered as “relevant” vs segment considered as “non-relevant”. Electroencephalography (EEG) represents a low-cost, non-invasive, and safe neurophysiological measurement tool to examine brain activity related to HVLA vertebral thrusts administration. EEG enables virtually real-time assessment of central nervous system changes induced by HVLA thrusts and has been used in previous studies examining the changes of HVLA vertebral thrusts directed at segments considered as “relevant”^8,9^. Numerous other studies have also used EEG in combination with somatosensory stimulation before and after HVLA thrusts directed at segments considered as “relevant”^8,10–13^. These studies found that HVLA thrusts applied to segments considered as “relevant” alter the amplitudes of several SEP peaks, in particular, the N20 and N30 peaks^8,10–13^. The most consistent change following HVLA thrusts directed at vertebrae considered as “relevant” is a reduction in the amplitude of the N30 SEP peak^8,10–13^.

Animal studies have clearly shown that the contact site for an HVLA thrust can have a significant effect on the magnitude of sensory input arising from muscle spindles in the paraspinal muscles^14^. However, it is also important to take into account the known alterations that occur to these paraspinal muscles and other tissues around a dysfunctional vertebral segment in humans, which may influence the neurophysiological effects of an HVLA thrust directed at a “relevant” vs “non-relevant” vertebra. There are, for example, known maladaptive plastic changes in the deep paraspinal muscles following a spinal injury in animal models^15–20^. Rapid atrophy^16,17^, muscle fibrosis, extensive fatty infiltration, changes in muscle fibre types^15,18–21^, and even changes to muscle spindles^22^ have all been found within the deep paraspinal muscles at various time-frames after a spinal injury in various animal models. Multiple studies in humans support such maladaptive plastic changes also occur in humans when their spines dysfunction or are injured^23–26^. These local paraspinal muscle changes coincide with 'smudging' within the primary sensorimotor cortices^27,28^ and have led scientists to conclude that disrupted or reduced proprioceptive signaling from deep paraspinal muscles likely plays a pivotal role in driving the long-term cortical reorganization and changes in the top-down control of the sensorimotor systems, and that this plays a vital role in driving the recurrence and chronicity of spinal pain^29^. With such clear evidence that maladaptive dysfunction of the deep paraspinal muscles can occur^15–20,22^, an HVLA thrust directed at such a dysfunctional vertebral segment that is surrounded by poorly functioning paraspinal muscles could produce a different physiological response compared to an HVLA thrust applied to a fully functioning vertebral segment with healthy paraspinal muscles and tissues. To test this hypothesis, the present study aimed to compare the changes in response to HVLA thrust directed to a vertebral segment considered as “relevant” with the changes in response to an HVLA thrust directed to a segment considered as “non-relevant”, using the most consistent neurophysiological measure, i.e., the N30 SEP amplitude, in adults with subclinical neck pain. We hypothesized that the group receiving HVLA thrust directed at a “relevant” vertebra would show a significant decrease in N30 SEP complex amplitude^8,10–13^.

## Methods

### Design and setting

This study was a double-blinded, randomized, active-controlled, parallel study conducted at the New Zealand College of Chiropractic, New Zealand. The study was approved by the New Zealand Health and Disability Ethics Committees (19/CEN/202), and the protocol was prospectively registered with the Australian New Zealand Clinical Trials Registry (ACTRN12620000175976, 17/02/2020). All participants gave written informed consent, which conformed to the Declaration of Helsinki.

### Participants

Participants were recruited through convenience sampling targeting the student and staff population at the New Zealand College of Chiropractic. Participants were included if they had a history of recurring and ongoing neck pain, aches, stiffness, or discomfort for which they had not sought treatment and were between the ages of 18 and 50 years. All participants were required to be pain-free at the time of the study. This requirement was intended to (1) avoid the confounding effect of current pain, (2) avoid any confounding effect from current or past treatment for more severe spinal problems and (3) ensure they were likely to actually need HVLA thrusts i.e., without a history of spinal problems there may not be any clinical reason to provide HVLA to their spines. Participants were excluded if they had no evidence of spinal dysfunction upon assessment by the chiropractor, had metal implants in their skull, had a history of severe neck pain (i.e., numeric pain rating scale ≥ 7/10), or had serious spinal pathology (i.e., malignancy, fracture, infection, hematoma, or cervical arterial dissection). If any of the participants had received previous chiropractic care for anything other than their neck pain, ache or tension, they were excluded if they had received HVLA thrusts within seven days of the day of data collection. Those who had received previous HVLA thrusts greater than seven days for anything other than their neck pain prior to data collection were not excluded.

Sample size calculations were made based on data from our unpublished pilot study wherein we investigated changes in SEPs before and after a single session of relevant HVLA thrust using a similar protocol. We calculated a required sample size of 84 using G*Power (version 3.1.9.4) based on the statistical t-tests (Means: Difference between two independent means [two groups]) to observe an Effect size d of 0.6212775 with α = 0.05, power β = 0.8, and an allocation ratio of 1. To compensate for  the dropouts, we recruited 96 participants.

### Randomization and blinding

Participants were allocated to either the “relevant” HVLA thrust or the “non-relevant” HVLA thrust group using an online randomization program (QMinim, Microsoft Corp., Redmond, WA, USA). A randomization sequence with 1:1 allocation was done with age (< 35 years or ≥ 35) and gender as a priori covariates. Participants and the investigators who collected or analyzed the data were blinded to group allocation, while the chiropractors who delivered the intervention were unable to be blinded due to the nature of the intervention. To ensure effective blinding of investigators, all recorded data were anonymized and given a code a priori. The investigators were kept blinded until the final analysis was completed.

### Study procedure

Following an initial screening, each participant’s cervical spine was assessed by a registered and experienced (> 10 years) chiropractor for the presence and site of spinal dysfunction/subluxation (i.e., “relevant” sites)^2^. Eligible participants came in for a single session comprising of SEP recording before and immediately after the HVLA thrust intervention. During the session, the participants were seated comfortably in a chair and were asked to focus their gaze on a fixed target on the wall and be relaxed to minimize the contamination of EEG signals.

### Somatosensory evoked potentials

SEPs were evoked by stimulating the median nerve of the dominant hand using electrical pulses from an electrical stimulator (Digitimer DS7AH, Hertfordshire, UK). After positioning the stimulating electrodes (Neuroline 700, AMBU A/S, Ballerup, DK) on the wrist, the intensity was slowly increased until the motor threshold, the lowest current intensity that produces a visible twitch of the thumb, was reached. A total of 1000 monophasic electrical pulses at a frequency of 2.3 Hz having 0.2 ms width were given to the median nerve.

### EEG

The EEG from 25 channels (frontal and frontal-central: FP1, FPz, FP2, F7, F3, Fz, F4, F8, FC5, FC1, FC2, FC6, AF7, AF3, AF4, AF8, F5, F1, F2, F6, FC3, FCZ, FC4, FT7, FT8) was recorded at a sampling rate of 2048 Hz using REFA amplifier (TMSi, Twente, NL). The ground electrode was placed at AFz. The electrode impedance was kept below 10 kΩ. During EEG recording, participants were asked to reduce eye blinks, eye movements and facial movements. Offline analysis of the EEG data was performed using custom scripts in MATLAB 2020a (The MathWorks, Inc., Natick, MA, USA) that utilised EEGLAB (version 2019)^30^, ERPLAB (version 2019)^31^, FieldTrip (version 20180912)^32^, and MATLAB functions^33^. At the start and end of the EEG recording, an additional 10 s of data was recorded to minimize the filtering artefacts in preprocessing. The standardized early-stage EEG processing pipeline (PREP) pipeline^34^ was utilized to determine faulty channels, discard line noise and acquire the average referenced data.

## Interventions

### HVLA thrust directed at cervical vertebrae considered as “relevant”

We defined the “relevant” HVLA thrust intervention as a high-velocity, low-amplitude thrust directed at a dysfunctional cervical spinal segment. The chiropractor determined each cervical spinal site to be “relevant” (i.e. dysfunctional) based on an examination which identified restricted intervertebral motion and pain provocation with motion palpation and palpable, asymmetric local hypertonic musculature, and any blocked or unusual joint play/end-feel of the spinal joints^1,35^. Participants in this group were eligible to have more than one site of dysfunction, yet only a single site was chosen for the HVLA thrust. In the instance of multiple sites of dysfunction, the chiropractor always defaulted to choose the more superior/cranial HVLA thrust to establish consistency with the HVLA thrust methodology.

**Figure 1:**
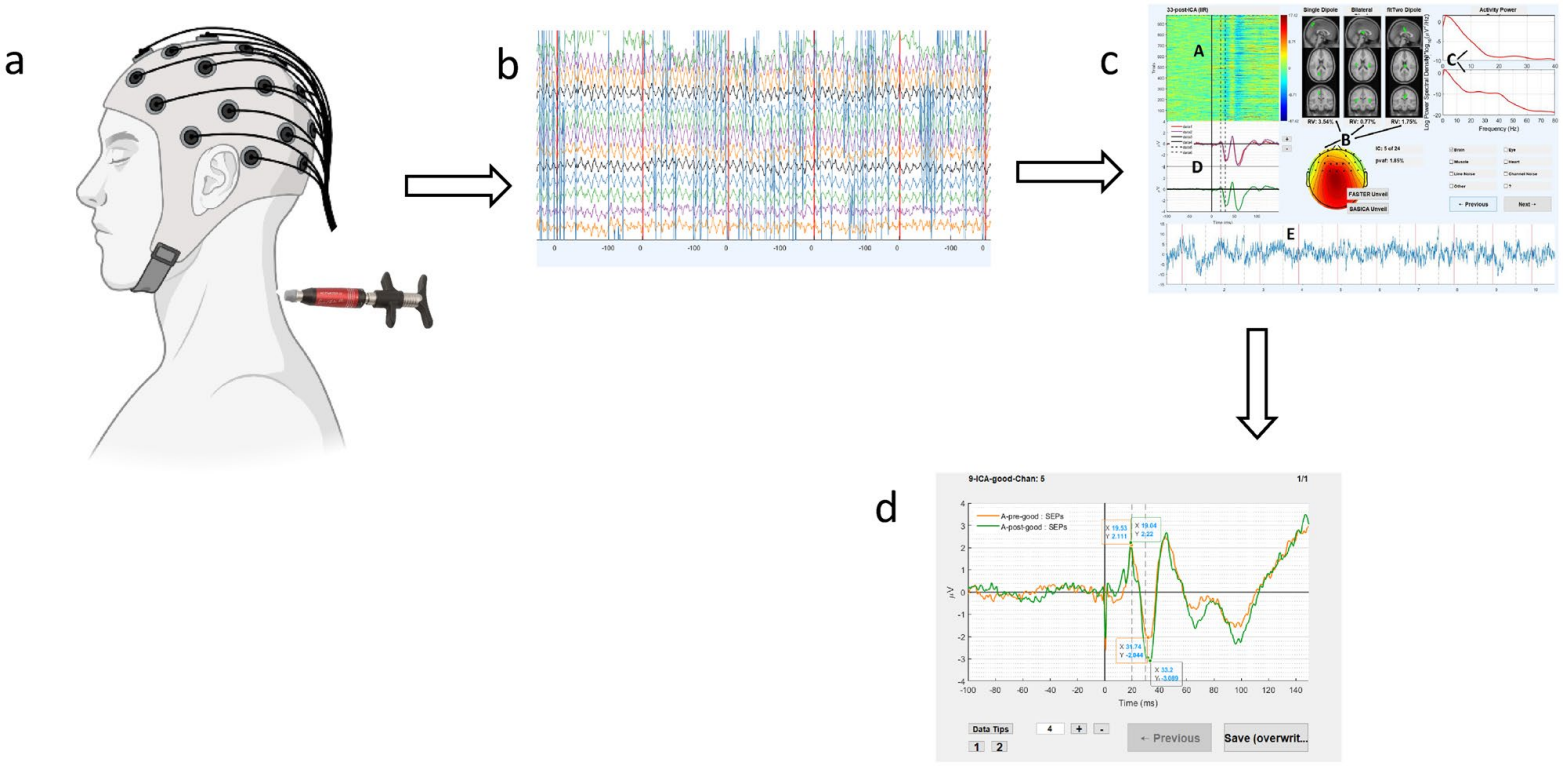
(**a**) Demonstration of the experimental setup involving the participant with EEG cap and the Activator instrument illustrated in the posterior cervical spine region. (**b**) Shows a section of the graphic user interface used for marking epochs. (**c**) Indicates graphic user interface (GUI) for accepting or rejecting independent components. (**d**) Graphic user interface for marking somatosensory evoked potentials.

The “relevant” HVLA thrust intervention involved the chiropractor delivering a single administration of HVLA thrust with an Activator instrument on the site of “relevant” cervical dysfunction (Fig. 1). The Activator instrument is a hand-held device that delivers fast, precise, and low-force thrust to the spine^36^. This device was preferred over manual delivery of HVLA thrust for the purposes of consistency with HVLA thrust administration, as its use would reduce the amount of variability in HVLA thrust parameters (i.e., force, amplitude, and duration).

### HVLA thrusts directed at cervical vertebrae considered as “non-relevant”

The “non-relevant” HVLA thrust intervention refers to a thrust directed at a non-dysfunctional spinal segment that had no signs of dysfunction upon examination by a chiropractor^1^. This intervention involved the delivery of a single HVLA thrust application via the Activator instrument. The non-dysfunctional vertebra targeted was always the one that was furthest away from the dysfunctional vertebra, yet within the cervical spinal region. In the event that there was more than one site of cervical dysfunction, the chiropractor avoided providing HVLA thrust at any site of dysfunction yet aimed to be furthest from the relevant segment. HVLA thrust was delivered by the same chiropractor who provided the relevant HVLA thrust intervention to ensure consistency.

Both groups received HVLA thrusts directed at a vertebra in the upper cervical spine. The main reason for this is that there are differences in the physiology of the upper and lower cervical spine that could introduce confounding variables that we wanted to avoid. Unlike the lower cervical spine, where muscles span multiple segments, the upper cervical spine has many small deep paraspinal muscles that cross individual spinal segments. Additionally, the deep paraspinal muscles of the upper cervical spine are rich in muscle spindles, which a crucial for mechanoreception and known to respond to the HVLA thrust. Therefore, to maintain as much consistency in the intervention level across groups the most cephalic segment was chosen for both groups, i.e., the most cephalic segment that either was or was not deemed dysfunctional.

### Data processing

Most of the GUIs and codes used were the same as in Navid et al.^8,33^. The mastoid channel ipsilateral to the dominant hand was used as the reference channel. The data was filtered with a 2nd order Butterworth band-pass filter with a frequency range from 1 to 500 Hz. SEPs were extracted from − 100 ms to 150 ms with respect to the stimulus. The pre-stimulus period was used for baseline correction. The post-stimulus period used was 150 ms and entails the cortex's response this study concentrates on. Contralaterally to the dominant hand, the trial-rejected, averaged, eyeblink-cleaned, and noise-cleaned event-related potential of either F3 for right-handed or F4 for left-handed participants was plotted and scanned for the components P22 and N30. This was done with a GUI for component marking.

### Statistical analysis

A linear mixed model was used to identify the effects of intervention site selection on the N30 amplitude. The intervention (“relevant” and “non-relevant”) and session (pre and post) were used as fixed factors. The between-paticipant variance was estimated using random intercept in the model. The models were implemented using lme4 package (version 1.1.23) in R (version 3.5.1)^37^. The pairwise comparisons were obtained using the emmeans package version 1.4.8^38^, adjusted for multiple comparisons using Tukey's honestly significant difference test. The significance threshold was set at *p* < 0.05.

## Results

**Figure 2:**
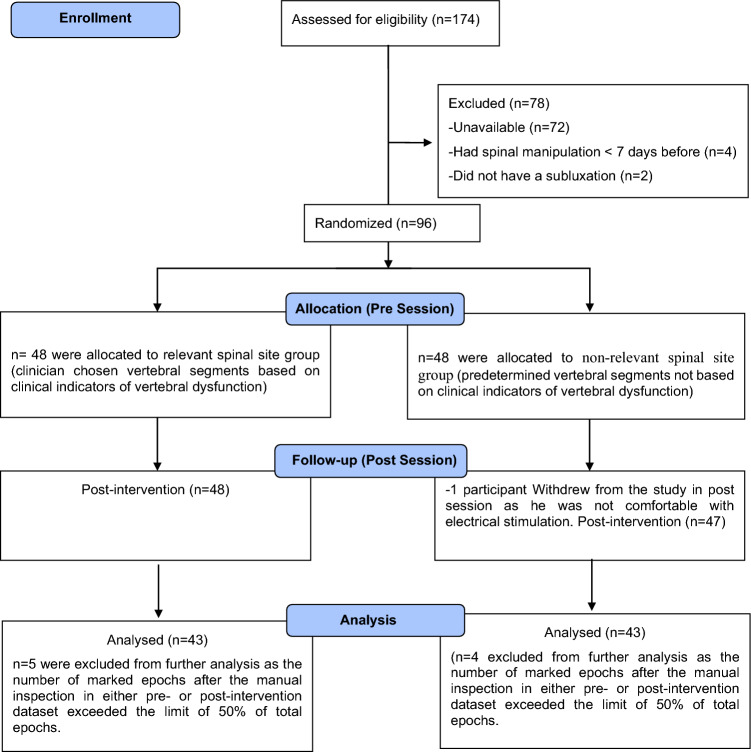
CONSORT study flow diagram.

Ninety-six participants met the eligibility criteria and were enrolled in the study between February 2020 and March 2020. The participant recruitment flow diagram is given in Fig. 2. The demographic characteristics of included participants in each group are given in Table 1.

**Table 1:** Demographic characteristics of participants in each group.

Variables	HVLA thrust applied to segments considered as “relevant”	HVLA thrust applied to segments considered as “non-relevant”
Gender		
Male, number (%)	20 (47)	17 (40)
Female, number (%)	23 (53)	26 (60)
Age, years (mean ± SD)	24.41 ± 5.05	24.83 ± 5.57

*SD* standard deviation.

### HVLA thrust site selection

The cervical spinal site of HVLA thrust administration in the “relevant” HVLA thrust group was most often C1 (n = 25), followed by C2 (n = 14) and C3 (n = 4). No patient in this group received HVLA thrust at a cervical spinal level caudal to C3. In the “non-relevant” HVLA thrust group, the most common site of HVLA thrust was C3 (n = 17), followed by C2 (n = 16), C1 (n = 8), C5 (n = 1), and C6 (n = 1). In the “non-relevant” HVLA thrust group, the maximum distance from the dysfunctional segment to the non-dysfunctional, manipulated segment was two vertebral segments.

### N30 complex amplitude

The mixed model showed a significant interaction between the site of intervention and session (F_1,84_ = 9.89, *p* = 0.002) (Table 2).

**Table 2:** Model results.

	F	Df	Df.res	Pr (> F)
Intervention	1.28	1	84	0.262
Session	3.06	1	84	0.084
Intervention: session	9.89	1	84	0.002

Pairwise comparisons (Table 3) revealed that there was a significant decrease in the N30 amplitude immediately after HVLA thrust was applied to segments considered as “relevant” (N30 complex difference % =  − 16.76 ± 28.32%, *p* = 0.005), whereas the N30 amplitude displayed a non-significant increase after HVLA thrust was applied to segments considered as “non-relevant” (N30 complex difference % = 19.58 ± 55.09%, *p* = 0.0757).

**Table 3:** Within-group differences based on estimated N30 amplitude from the statistical model.

Contrast	Estimate ± SE	95% CI	t. ratio	*p*-value
“Non-relevant” post – “non-relevant” pre	0.12 ± 0.12	[− 0.19, 0.43]	0.99	0.757
“Relevant” post – “relevant” pre	− 0.41 ± 0.12	[− 0.72, − 0.10]	− 3.46	0.005

**Figure 3:**
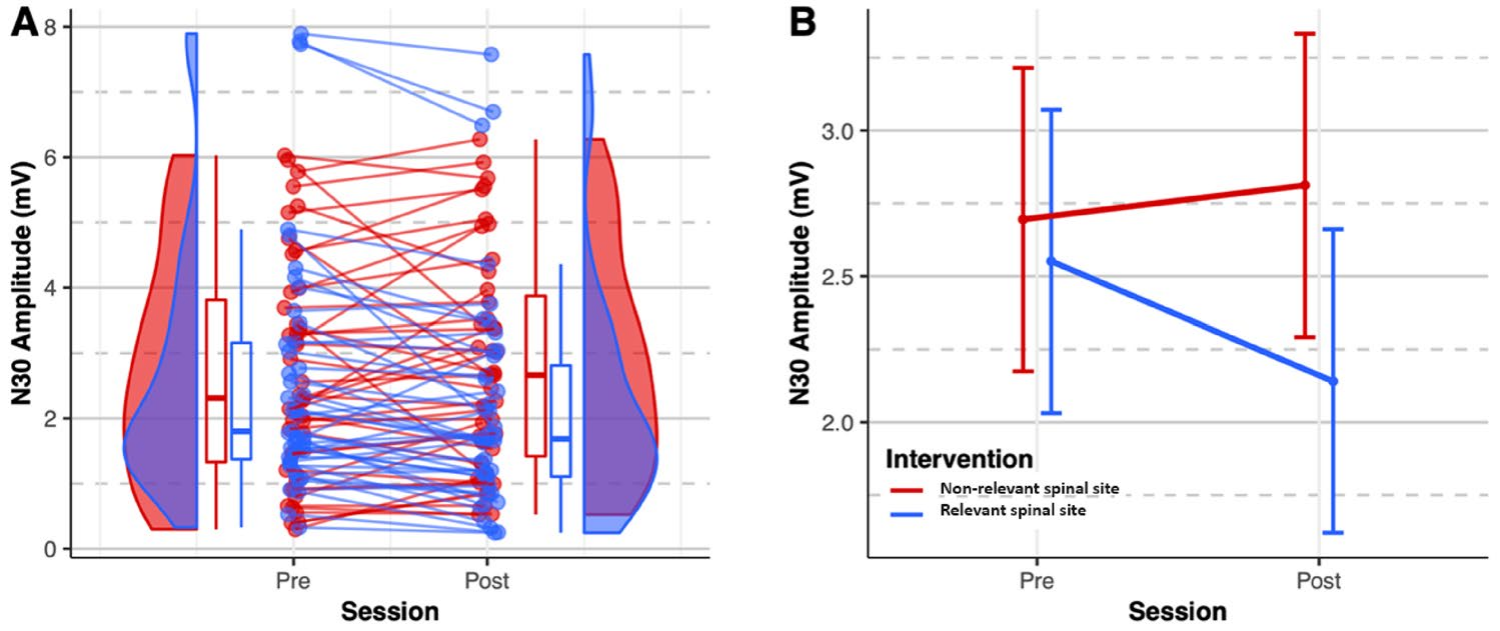
N30 amplitude. (**A**) Dots represent the N30 amplitude of individual subjects. Boxplots show the median, 25th and 75th percentiles. The distribution plots show the density distribution estimated by a Gaussian kernel with an SD of 1.5. (**B**) The error bars represent the estimated mean ± 95% CI from the statistical model.

Figure 3 shows the distribution and differences of the N30 amplitude before and immediately after the interventions such that the N30 amplitude significantly decreased immediately after HVLA thrust was applied to segment considered as relevant.

## Discussion

This randomized controlled trial was the first study to compare the immediate changes in response to the application of an HVLA thrust at a cervical vertebra considered as “relevant” versus an HVLA thrust directed at a cervical vertebra considered as “non-relevant” on human brain activity as measured by the N30 SEP complex amplitude. The present study found a significant decrease in the N30 SEP complex amplitude (about − 17%) immediately after an HVLA thrust applied to a cervical segment considered as “relevant” (i.e., a dysfunctional segment), whereas a non-significant increase in the N30 SEP complex amplitude was recorded immediately after an HVLA thrust applied to a cervical segment considered as “non-relevant” (i.e., a non-dysfunctional segment). According to previous research, a decrease in the N30 SEP complex amplitude is suggestive of changes in sensorimotor function occurring within the prefrontal cortex^12^. Therefore, the current study findings suggest that applying the HVLA thrust at a dysfunctional segment may induce greater level of sensorimotor integrative changes than applying an HVLA thrust at a non-dysfunctional site.

### Comparison with the literature

The present study adds to the limited research regarding HVLA thrust site selection^39^. Studies using animal models have demonstrated one can maximize proprioceptive afferent inputs and muscle spindle responses coming from a specific spinal segment when HVLA thrusts are directed at that same spinal segment with velocities greater than 20–30 mm/s and with thrust rates greater than 300N/s^14,40^. In one animal study, the authors created spinal dysfunction with a facet fixation model^41^ and found that such spinal fixations altered paraspinal sensory responses during “relevant” HVLA thrusts at these levels. However, there is paucity of evidence of differences in neurophysiologic changes in humans following HVLA thrust applied to segments considered as “relevant” versus HVLA thrust applied to segments considered as “non-relevant”. The current study, therefore, adds to the limited research regarding HVLA thrust site selection in the cervical spine in humans. The present study has the potential to offer insights into practical decision-making for chiropractors when choosing between HVLA thrust at one cervical site versus another. The current findings support that chiropractors' selection of which cervical site to apply HVLA thrust may be relevant with respect to subsequent sensorimotor changes. A recent systematic review reported that spinal manipulation applied to suspected relevant sites did not yield a superior result for clinical outcome measures compared to spinal manipulation applied to non-relevant sites in nine of 10 included studies^39^. However, the study was limited in that the included studies demonstrated variability in what constituted a relevant site of spinal manipulation, and in some instances, both the treatment and control groups received a potentially relevant HVLA thrust. In three of the included studies^42–44^, two different HVLA techniques were compared, yet potentially targeted dysfunctional, hypomobile, or symptomatic spinal segments, resulting in improved pain and disability outcomes. Although the between-group HVLA techniques varied, the segment(s) to which HVLA thrust was applied could represent relevant sites^42–44^.

Two of the nine studies^45,46^ compared two groups receiving HVLA thrust manipulation of spinal levels pre-determined by the experimental design rather than clinical context. For example, Romero del Rey et al.^45^ compared HVLA manipulation of either C1-C2 with HVLA thrust manipulation C3-4, C7-T1 and T5-6, while Bautista-Aguirre et al.^46^ compared HVLA thrust manipulation of C7 with HVLA manipulation of T1. In both of these studies, the sites of HVLA thrusts were therefore not chosen according to patient-specific clinical findings; hence all groups potentially received non-relevant HVLA manipulations^45,46^. In these studies, there were no significant between-group differences in pain outcome scores, pressure pain thresholds, or upper extremity grip strength.

Four of the nine studies^47–50^ tested whether applying an HVLA thrust to the most tender lumbar^47,48^ or cervical^49^ segment, or hypomobile segment based on end play assessment^50^ (i.e., HVLA thrust on “relevant” vertebral segment would lead to better clinical outcomes compared to applying a general HVLA thrust at a pre-determined cervical or thoracic spinal segment (i.e., HVLA thrust on “non-relevant” vertebral segment). In all four of these studies, there were no differences in pain scores (pain intensity, pressure pain threshold, disability and global perceived change) or stiffness after the relevant HVLA lumbar or cervical segment manipulation compared with the non-relevant HVLA thoracic manipulation^47–49^ or cervical manipulation^50^. For all HVLA groups, the pain measures improved^47–49^. In the tenth study^51^, the authors compared an HVLA thrust aiming to improve the mobility of a dysfunctional (hypomobile) thoracic segment with a less specific thoracic HVLA manipulation provided in the direction of normal mobility. In this study, the relevant thrust significantly reduced cervical pain compared to the non-relevant HVLA manipulative thrust^51^. Given the above limited and conflicting findings, it remains unclear whether it is therapeutically important to aim to apply HVLA thrust to a “relevant” or “non-relevant” vertebral site when treating spinal pain or dysfunction.

Previous measures used in studies examining the relevance of HVLA thrusts site selection have had inherent limitations. The most commonly measured outcomes, such as pain intensity, range of motion, or disability, may be influenced by patient expectations or may not be expected to change with only a single application of HVLA vertebral thrusts^39^. Few studies have used imaging tests to examine this question, and while study designs using radiography to examine changes in intervertebral motion may be useful^52^, these are hindered by the necessity of exposure of participants to ionizing radiation. Newer magnetic resonance imaging studies (e.g. examining disc diffusion) are promising^53^ but may be limited by cost.

### Possible mechanisms

The present findings provide evidence that cervical HVLA thrust directed to a vertebral segment considered as “relevant” produces distinct neurophysiological changes evident via EEG via decreased N30 SEP complex amplitude. Previous studies have shown decreases in N30 SEP peak amplitudes following relevant HVLA thrust in subclinical spinal pain populations^10,11,54^. This amplitude reduction is attributed to changes in somatosensory processing at the cortical level, particularly within the prefrontal cortex^12^. Other neural generators of the N30 amplitude complex include the primary sensory cortex, basal ganglia, thalamus, premotor areas, and primary motor cortex^55–61^. The frontal N30 peak is thought to reflect early sensorimotor integration^62,63^.

Drawing from the insights in the literature on animal fixation model^41^, where spinal fixations were observed to modify sensory responses in paraspinal muscles during “relevant” HVLA thrusts, along with our current findings, we infer that the neurophysiological mechanisms associated with these “relevant” HVLA thrusts likely involve the activation of these paraspinal proprioceptive sensory responses, particularly in the presence of dysfunction. Paraspinal tissue dysfunction has, as discussed earlier, been noted to occur following a spinal injury^15–20^. This paraspinal tissue dysfunction includes rapid atrophy of deep paraspinal muscles^16,17^, deep paraspinal muscle fibrosis, extensive fatty infiltration of such muscles, changes in muscle fibre types within such muscles^15,18–21^ and even changes to muscle spindles themselves within the deep paraspinal muscles at the injured segment^22^. These local paraspinal muscle changes coincide with 'smudging' within the primary sensorimotor cortices^27,28^ and have led scientists to conclude that disrupted or reduced proprioceptive signalling from deep paraspinal muscles likely plays a pivotal role in driving the long-term cortical reorganisation and changes in the top-down control of the sensorimotor systems and that this plays a vital role in driving the recurrence and chronicity of back pain^29^. As applying an HVLA thrust is known to activate muscle spindles in surrounding paraspinal muscles^14,40^, it, therefore, seems plausible that applying the HVLA thrust at such dysfunctional segments could result in different clinical effects, as compared to applying an HVLA manipulation to a healthy vertebral segment, with non-fibrotic, non-fatty infiltrated paraspinal segments. The current study would support this notion.

### Clinical and research implications

The decrease in N30 amplitude noted in the present study suggests that the neurological activity within the network responsible for generating the N30 component (i.e., basal ganglia, thalamus, premotor cortex, prefrontal cortex and motor-cortex) decreases in response to HVLA thrust applied to cervical segment considered as “relevant”^55–61^. However, in light of the findings by Lelic et al.^12^ that showed when relevant HVLA thrusts were applied to research participants, the decrease in the N30 SEP complex amplitude appeared to reflect mainly changes in sensorimotor function occurring within the prefrontal cortex, the decrease in the N30 SEP complex amplitude found in the current study most likely also reflects alterations in sensorimotor function within the prefrontal cortex. Accordingly, relevant HVLA thrust may influence sensorimotor integration and related neuromuscular functions. Future studies can explore the clinical relevance of these findings.

### Strengths, limitations and future research

A strength of the study was the large sample size which supports the fact that is unlikely that the observed reduction in N30 complex amplitude in the relevant HVLA thrust group occurred by chance. Limitations of the study include that a single cervical HVLA thrust application may not reflect the long-term care performed in clinical practice. However, this protocol design was intended to avoid repeatedly exposing the “non-relevant” HVLA thrust group to HVLA thrust directed at the non-dysfunctional segment for a longer period. Another limitation is that the changes in N30 SEP complex amplitudes were only measured immediately after one thrust application and not several time periods after the HVLA thrust application. Therefore, it is unclear how long the effects on N30 complex amplitude last after the HVLA thrust.

There are several potentially important differences between spinal regions that preclude our ability to generalize the current study findings to other regions of the spine (i.e., thoracic, lumbar, or sacroiliac joints). Importantly, the density of mechanoreceptors is higher in the cervical region^64,65^. HVLA thrust is known to activate muscle spindles in surrounding paraspinal muscles^14,40^. Therefore, mechanoreceptors such as muscle spindles are believed to sense the impulse provided by HVLA thrust and trigger subsequent neurophysiological changes^1^ and thus may produce greater responses, as evident via EEG. The height of cervical vertebrae and, therefore, the corresponding motion segments are smaller, thus potentially requiring a greater degree of precision or lower degree of force application with HVLA thrust. There is also a difference in innervation; for example, cranial nerve five has an anatomical relationship to the upper cervical spine and may be implicated in cases of neck pain^66^, whereas cranial nerves are unrelated to the other spinal regions. The current study should therefore be replicated in other regions of the spine to determine if the findings are consistent or dependent upon the spinal region.

The results of this study may not be generalizable to other methods of HVLA thrust administration. As the current study used an instrument to administer HVLA thrust (Activator), the delivery of force could be both lower in magnitude and/or more localized to a specific vertebrae or motion segment. While little is known about this topic, it is possible that other forms of HVLA thrust applied manually could lead to a broader biomechanical effect on the spinal target^53^ and potentially a less predictable neurophysiological response. However, in the current study, the HVLA thrust delivered via the Activator instrument led to a significant decrease in the N30 SEP peak complex, similar to what has been found following HVLA thrusts delivered manually as well^8,10–13^. In these previous studies, HVLA thrusts were likewise targeted at dysfunctional segments, thus representing HVLA thrusts. The current study did not measure several clinical variables, such as baseline or post-HVLA thrust changes in pain severity or range of motion. Accordingly, it is not known if the observed changes in N30 amplitude correlate with measures of clinical improvement such as reduced pain, pain pressure thresholds, reduced stiffness, or improved mobility. Future studies could replicate the present design while including clinical outcomes alongside EEG. In addition, a longitudinal design with multiple HVLA thrust interventions may enable the examination of potential long-term or progressive changes in neurophysiological measures.

The present study may only be generalizable to younger adults as the mean age for each group was 24 to 25 years. It is unknown if age-related degenerative changes in the cervical spine would interfere with mechanoreception related to HVLA thrust or the observed subsequent neurophysiological changes. In addition, the current study may not be generalizable to people with severe neck pain, such as cervical radiculopathy or disc herniation, as only individuals with milder symptoms were included. The current study could be replicated in an older population or individuals with more severe neck pain syndromes for comparison.

Regarding the blinding of the chiropractor providing the HVLA thrust, it could be noted that an alternative approach could have involved a different chiropractor conducting the assessments on all participants and subsequently informing the treating chiropractor about the specific site for the HVLA thrust without disclosing its relevance. However, for the sake of maintaining consistency and minimizing potential variability in technique application, the same chiropractor was deliberately chosen to perform both the assessments and the HVLA thrust intervention. This decision was further supported by the utilization of standardized instrument-assisted thrusting with Activator, which aimed to reduce potential sources of variation. Despite this, it is important to acknowledge that not blinding the treating chiropractor may introduce a source of bias. In order to mitigate potential bias, we took measures to blind both the participants and the assessors involved in the study. This additional step was implemented to enhance the rigor and integrity of our methodology.

## Conclusion

This randomized controlled trial was the first to investigate the immediate changes in response to an HVLA thrust site selection in the cervical spine using a neurophysiological EEG outcome measure and found evidence that HVLA thrust directed at a cervical site considered as dysfunctional significantly reduces N30 amplitude immediately after such intervention. In contrast, HVLA thrust directed at a cervical site considered as non-dysfunctional causes no significant change. The present findings suggest that clinicians' selection of where to apply cervical HVLA thrust is likely to be relevant with regards to affecting the subsequent sensorimotor response. Further research is needed to correlate these changes with clinical outcomes, repeat the study design in other spinal regions and patient populations, and examine both potential changes at short, medium and long terms, as well as the longitudinal response to multiple HVLA thrust sessions.

## Data Availability

The numerical data supporting the conclusions of this article will be made available by the authors without undue reservation. Interested individuals can obtain this data by making a reasonable request to the corresponding author, Imran Khan Niazi. The Local Ethics Committee adhering to local data protection laws, does not allow the sharing of individuals’ raw data.
